# Retropharyngeal Blastomycosis Abscess Causing Osteomyelitis, Discitis, Cervical Deformity, and Cervical Epidural Abscess: A Case Report

**DOI:** 10.7759/cureus.45570

**Published:** 2023-09-19

**Authors:** Faraaz Azam, William H Hicks, Mark N Pernik, Kathryn Hoes, Russell Payne

**Affiliations:** 1 Neurological Surgery, University of Texas Southwestern Medical Center, Dallas, USA; 2 Neurosurgery, University of Tennessee Health Science Center, Memphis, USA; 3 Neurological Surgery, University of Pittsburgh Medical Center, Pittsburgh, USA

**Keywords:** epidural abscess, cervical osteonecrosis, cervical spine infection, retropharyngeal abscess, blastomycosis

## Abstract

Blastomycosis infection is caused by the inhalation of the spores of the dimorphic *Blastomyces *sp.fungus. While more commonly a self-limited infection of the lungs, extrapulmonary manifestations arise from hematogenous or contiguous spread. Disseminated infection most often includes skin lesions and osteomyelitis; however, central nervous system (CNS) involvement is infrequently reported in the literature. Herein, we present a case of a retropharyngeal blastomycosis abscess leading to cervical spine osteonecrosis with retropulsion, deformity, and a spinal epidural abscess, and we discuss the relevant literature. The patient was successfully treated with cervical traction, followed by a combined anterior-posterior cervical approach, including abscess drainage, corpectomies, and instrumented fixation. Postoperatively, the patient completed 12 months of voriconazole and had near resolution of preoperative symptoms. Expediting neurosurgical intervention, such as the utilization of decompression, the clearance of infectious burden, and the correction of alignment, is critical for preventing downstream complications. Retropharyngeal blastomycosis abscesses are rare, and we report one of the rare instances of dissemination to and the degeneration of the cervical spine.

## Introduction

Blastomycosis is an endemic fungal infection caused by the inhalation of *Blastomyces *spp. spores. *Blastomyces *spp. typically infect immunocompetent hosts resulting in self-limited pulmonary disease [1,2]; however, 30%-50% of cases are asymptomatic or subclinical [3]. Pulmonary manifestations include a dry cough, dyspnea, fever, chills, and night sweats. Severe, disseminated infections more commonly occur in immunocompromised patients [1,4]. The most common extrapulmonary sequelae of blastomycosis are verrucous and ulcerative dermatitis, osteomyelitis, septic arthritis, and genitourinary infection [5]. Blastomycosis in the central nervous system (CNS) is rare, but it is associated with increased morbidity and mortality risk due to a lack of systemic disease burden and a protocol describing the optimal treatment [6].

In the literature, CNS blastomycosis manifests as meningitis, intracranial abscess, and spinal epidural abscesses, mainly in immunocompromised patients [6-9]. The most common presentation was headaches related to meningitis or an intracranial mass lesion [6]. Patients with intracranial mass lesions and delayed or interrupted treatment had elevated mortality rates compared to those with early and consistent treatment [6,7].

In cases with CNS involvement, there is usually concomitant infection at non-CNS sites. However, there have been limited documented reports of cervical spine dissemination. Herein, we describe the clinical course and treatment of an immunocompetent patient with a retropharyngeal blastomycosis abscess with associated cervical osteomyelitis and cervical spinal epidural abscess.

## Case presentation

Patient presentation

A 48-year-old immunocompetent male was transferred to our care for an acute exacerbation of neck pain, dyspnea, and odynophagia. The patient initially reported to the emergency department with a two-month history of acute or chronic neck pain and 63 pounds of weight loss. He described new dyspnea and dysphagia starting 10 days prior. He then developed left upper extremity pain that worsened over the preceding week. His past medical history included 30 years of neck pain, regular cigar usage, benign prostatic hyperplasia, obstructive sleep apnea, asthma, and deep vein thrombosis. Notably, the patient denied any recent travel history. Relevant physical examination findings included increased work of breathing and left upper extremity monoparesis. Strength was 4 out of 5, with no changes in sensation. Initial laboratory results revealed elevated WBCs, C-reactive protein (CRP), and erythrocyte sedimentation rate (ESR).

Imaging

**Figure 1:**
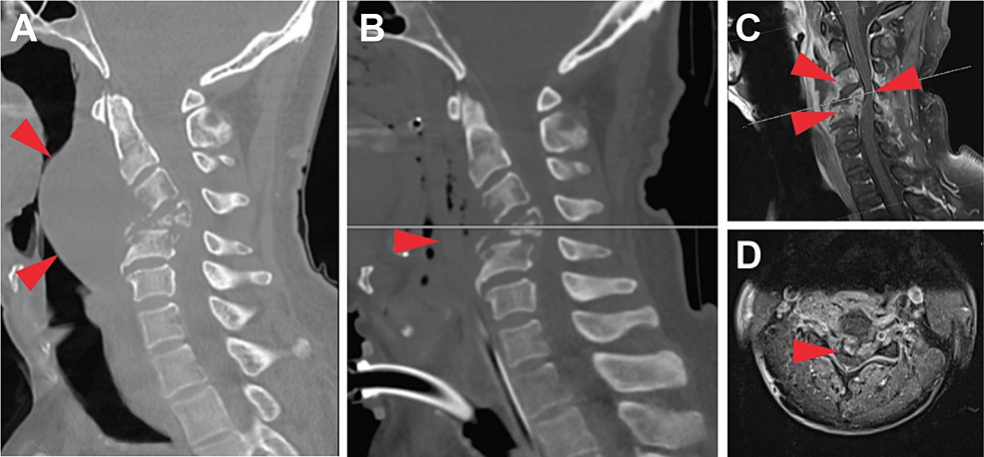
CT and MRI findings during the initial workup and management. (A) Sagittal CT demonstrates sizeable prevertebral collection. Posteriorly, there is cervical spinal canal stenosis due to extensive vertebral body destruction and retropulsion. (B) CT following tracheostomy and pharyngotomy shows a substantial decrease in the size of the prevertebral collection. (C and D) Sagittal and axial post-contrast MRI highlights extensive vertebral body enhancement, bony destruction, cervical spinal stenosis, and spinal cord compression. CT, computed tomography; MRI, magnetic resonance imaging

Plain radiographs of the cervical spine demonstrated a lytic process involving the anterior aspect of C4 and C5, leading to increased kyphosis, severe spondylosis, and spinal stenosis. There was 3.7 cm of inflammation in the prevertebral soft tissue, causing the narrowing of the oropharynx. A computed tomography (CT) of the cervical spine supported these findings and revealed 7 mm of retrolisthesis of C4 on C5 (Figure 1A and Figure 1B). Further imaging found a mucosal thickening of the paranasal sinuses and the opacification of the left and right nasal cavities, nasopharynx, oropharynx, hypopharynx, supraglottis, and glottis. Soft tissue edema was again demonstrated, spanning from C2 to C5. The spinal canal size was measured to be 5 mm in the anteroposterior dimension at C4. The post-tracheostomy magnetic resonance imaging (MRI) revealed a potential rupture of the anterior longitudinal ligament with multilevel degenerative disc disease of the cervical spine and facet arthropathy (Figure 1C and Figure 1D). There was an intermediate hypointense/short tau inversion recovery (STIR) hyperintense signal abnormality of the C3, C4, and C5 vertebrae with contrast enhancement. These findings extended along the lateral aspect of C2, right pedicles, and lateral masses of C3 and C4.

Initial management

Based on the above findings, there was a concern for atypical osteomyelitis versus malignancy. A nasogastric tube was placed, and laryngoscopy was performed to explore a potential retropharyngeal abscess. Secretions in the naso- and oropharynx were aspirated. There were no notable defects in the posterior pharyngeal wall. Posterior midline and right anterior pharyngotomies were performed by otolaryngology, from which 40 mL of cloudy, purulent fluid was removed. Due to the extensive cervical spine involvement, the patient was transferred to neurosurgery.

Neurosurgical treatment

Given the prevertebral abscess, anterior epidural abscess, osteodiscitis, osteonecrosis, retrolisthesis of the C4 and C5 vertebral bodies with significant retropulsion, upper cervical kyphosis, and cord impingement, a staged anterior-posterior approach was planned. He was first placed in cervical traction at the bedside to assist with correcting the cervical spine deformity. Otolaryngology was then consulted to assess for a persistent connection between the pharynx and the prevertebral space following the previously performed pharyngotomy. There was no apparent communication between the pharynx and the prevertebral space. The following day, he was taken to the operating room for the decompression and stabilization of the cervical spine.

The anterior approach (stage I) was performed to drain the abscess and obtain cord decompression with discectomies and corpectomies. After fluoroscopic localization, an incision was created medial to the sternocleidomastoid from the mandible to the level of the cricoid cartilage at the tracheostomy site. The platysma was dissected to identify and divide the omohyoid. Further dissection revealed a phlegmon-filled capsule covering the prevertebral fascia from which culture specimens were sent. Discectomies were performed at C3-C4, C4-C5, and C5-C6. We removed retropulsed bone fragments at C4, followed by irrigation and drainage. Additionally, corpectomies were performed on the C3-C5 vertebral bodies. An expandable stratosphere interbody cage was placed and secured to the C2 and C6 vertebrae via an anterior cervical plate with additional bone graft material placed inferior to the cage.

Next, the patient was transitioned to prone for the posterior approach (stage II). All laminae from C2 to T2 were exposed and prepared for posterolateral arthrodesis. Pedicle screws were placed bilaterally on T1 and T2, lateral mass screws were placed at C3-C6, and pars screws were placed at C2. Rods were then placed and secured. After checking alignment and setting the screws to their final position, Clorpactin was used to irrigate the field, followed by antibiotic saline. There were no intraoperative changes in neuromonitoring, and the patient tolerated the procedure well.

Infectious disease and pathology reports

An infectious workup was performed on the samples from the prevertebral fascia during the anterior approach.* Blastomyces dermatitidis* was confirmed with fungal culture and microscopy showing wide-based budding. There were signs of acute and chronic inflammation, with multinucleated giant cells in the prevertebral tissue samples. Simple and budding yeast was seen, indicating active metabolism. Biopsies from the C4 and C5 vertebrae confirmed osteomyelitis and granulomatous inflammation. Infection and necrosis of the left longus muscle were also noted. There was no evidence of neoplastic tissue in any sample.

Postoperative course

**Figure 2:**
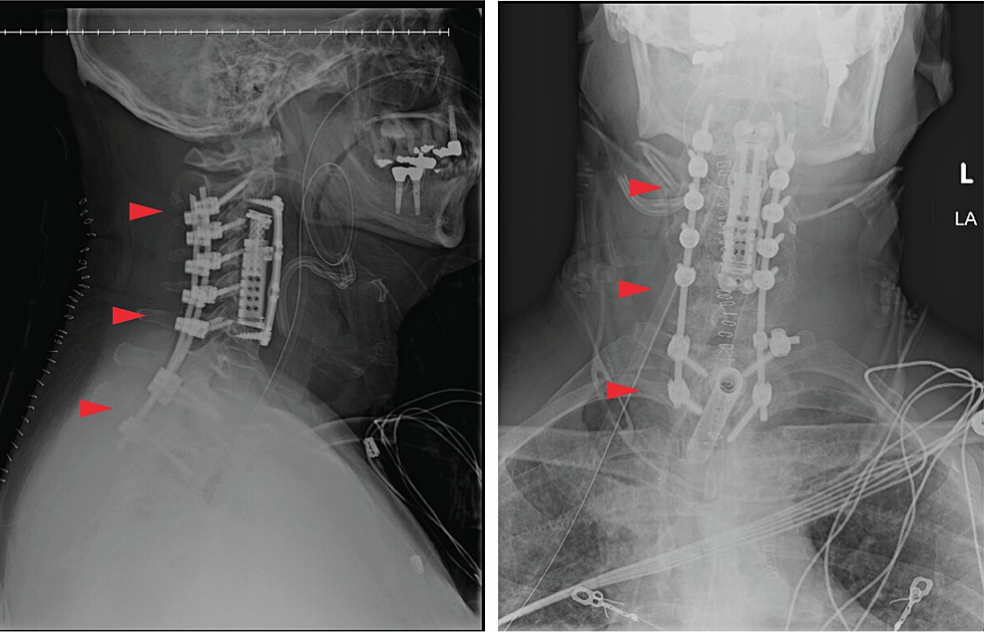
Inpatient postoperative radiographs demonstrating hardware alignment.

The patient was discharged on hospital day 14 with improved neck pain, dysphagia, and odynophagia. Postoperative radiographs showed the stable alignment of all screws and implants (Figure 2). The patient was transferred back to his local facility with an initial antifungal regimen of liposomal amphotericin B. He was then subsequently discharged and transitioned to posaconazole and then itraconazole, both complicated by hypokalemia. A final prescription of voriconazole was well tolerated and prescribed for a course of one year. At a one-year follow-up, the patient denied any neck pain, radicular pain, dysphagia, or symptom recurrence. Additionally, a CT of the cervical spine showed postoperative findings consistent with the procedures performed, hardware alignment, and unremarkable paraspinal soft tissue.

## Discussion

Blastomycosis is a fungal infection that involves the inhalation of the spores of *Blastomyces dermatitidis* and other subspecies. It is rarely associated with disseminated infection in immunocompetent individuals, and most cases present with self-limited, localized respiratory infections. CNS involvement is exceedingly rare, occurring in only 5%-10% of cases [6]. Our documented case is one of the rare incidences of disseminated blastomycosis to the cervical vertebrae and cervical epidural space with extensive bony destruction and cervical instability in an immunocompetent individual.

While the initial otolaryngology evaluation found no apparent defect in the posterior oropharynx, it is difficult to ascertain whether the infection arose primarily in the cervical vertebrae or spread to the prevertebral space from adjacent tissue. The spread of the infection to the cervical spine likely occurred from a retropharyngeal abscess that extended through a prevertebral defect into the spinal column. Blastomycoses have been noted to occur more frequently in smokers, as was the case with our patient, due to upper respiratory tract irritation and mucosal damage [10]. The most common etiologies of retropharyngeal abscesses are bacteria such as *Streptococcus pyogenes*, *Staphylococcus aureus*, and other respiratory organisms [11]. Due to the varied etiology, the treatment of retropharyngeal abscesses is pathogen-specific. However, due to the potential for airway obstruction, treatment routinely requires otolaryngology for the surgical drainage and maintenance of a patent airway.

Due to the rarity of vertebral dissemination, the available evidence is largely limited to case reports. While an immunocompromised status is a risk factor, most reported cases of CNS vertebral blastomycosis occurred in immunocompetent individuals [12-19]. This is unlike other typical fungal pathogens, as most infections are opportunistic. Other notable risk factors include diabetes and HIV. In a case series of vertebral blastomycosis, Saccente et al. reported a contiguous abscess in all of their 12 patients [16]. The cases primarily consisted of thoracolumbar infections, with contiguous paravertebral and psoas abscesses also present. Other cases of cervical blastomycosis are mentioned in the literature; however, an extensive discussion of the clinical course and operative considerations, notably airway obstruction and the correction of cervical deformity, has not been well documented [16]. As in our patient, the treatment of severe disseminated blastomycosis begins with liposomal amphotericin B for several weeks, with a transition to azole therapy for a minimum of one year [6,16]. With aggressive and continued treatment, good recovery has been seen, with no significant long-term deficits [20].

## Conclusions

We detail a unique case of blastomycosis infection of the cervical spine with the infiltration of the epidural space associated with a retropharyngeal abscess. In cases characterized by infectious osteonecrosis leading to neurological deficits and cervical deformity, prompt neurosurgical intervention to decompress the spinal cord, correct alignment, and decrease the infectious burden is critical. A fungal etiology should be considered, even without travel to an endemic region or an apparent defect in the posterior oropharynx. Additionally, the combination of cervical deformity and significant retropharyngeal inflammation creates a difficult airway, which necessitates prompt multidisciplinary care with otolaryngology colleagues. The awareness of potential fungal etiologies would allow for a quicker diagnosis and treatment, reducing long-term consequences and disease exacerbation.
